# Heterogeneity of regional and national hospitalization burden of lupus nephritis and systemic lupus erythematous

**DOI:** 10.1093/ckj/sfaf162

**Published:** 2025-07-01

**Authors:** Alejandro Avello, Raúl Fernández-Prado, Daria Abasheva, Ignacio Mahillo, Miguel Ángel González-Gay, Catalina Martín-Cleary, José Miguel Arce-Obieta, María Vanessa Pérez-Gómez, Beatriz Fernández-Fernández, Alberto Ortiz

**Affiliations:** Nephrology and Hypertension, IIS-Fundacion Jimenez Diaz UAM, Madrid, Spain; RICORS2040, Madrid, Spain; Nephrology and Hypertension, IIS-Fundacion Jimenez Diaz UAM, Madrid, Spain; RICORS2040, Madrid, Spain; Nephrology and Hypertension, IIS-Fundacion Jimenez Diaz UAM, Madrid, Spain; RICORS2040, Madrid, Spain; Statistics, IIS-Fundacion Jimenez Diaz UAM, Madrid, Spain; Rheumatology, IIS-Fundacion Jimenez Diaz UAM, Madrid, Spain; Nephrology and Hypertension, IIS-Fundacion Jimenez Diaz UAM, Madrid, Spain; RICORS2040, Madrid, Spain; Department of Medicine, School of Medicine, Universidad Autónoma de Madrid, Spain; Health Information and Clinical Coding Department, IIS-Fundacion Jimenez Diaz UAM, Madrid, Spain; Health Information and Clinical Coding Department, IIS-Fundacion Jimenez Diaz UAM, Madrid, Spain; Nephrology and Hypertension, IIS-Fundacion Jimenez Diaz UAM, Madrid, Spain; RICORS2040, Madrid, Spain; Department of Medicine, School of Medicine, Universidad Autónoma de Madrid, Spain; Nephrology and Hypertension, IIS-Fundacion Jimenez Diaz UAM, Madrid, Spain; RICORS2040, Madrid, Spain; Department of Medicine, School of Medicine, Universidad Autónoma de Madrid, Spain; Nephrology and Hypertension, IIS-Fundacion Jimenez Diaz UAM, Madrid, Spain; RICORS2040, Madrid, Spain; Department of Medicine, School of Medicine, Universidad Autónoma de Madrid, Spain

**Keywords:** cost, epidemiology, hospitalization, lupus nephritis, systemic lupus erythematosus

## Abstract

**Background:**

Differences between regional healthcare systems in the in-hospital burden and care of systemic lupus erythematosus (SLE) and lupus nephritis (LN) are poorly characterized. Their analysis may provide benchmarking opportunities that improve the quality and sustainability of care.

**Methods:**

We retrospectively investigated the hospitalization burden of SLE and LN in 2019–2021 across Spanish regional healthcare systems using the Spanish National Hospital Discharge Records database (RAE-CMBD) and National Statistics Institute (INE) data.

**Results:**

Of 66 262 724 hospitalization episodes from 644 public and private hospitals, 10 781 had a primary diagnosis of SLE, of which 2481 (23%) were for LN. The mean annual nationwide hospitalization case incidence was 70.61 and 1.75 per 100 000 population for SLE and LN, respectively. Regional differences were large: 48.0-fold and 6.9-fold between regions with the highest and lowest incidence for SLE and LN, respectively. In multivariate analysis, net household income and percentage of foreign-born population were associated with the number of SLE and LN hospitalization episodes. Internal medicine managed 28% of SLE and 15% of LN hospitalizations, nephrology 14% and 56% and rheumatology 23% and 11%, respectively, but there were large regional differences. The mean SLE and LN stays were 8.85 and 6.92 days (5.47 and 5.41 for nephrology and 11.18 and 11.83 for internal medicine), respectively. The average all patient refined diagnosis related groups (APR-DRGs) cost per episode was €2408 for SLE and €3563 for LN. The average yearly costs were €167 985 per million population (pmp) for SLE hospitalizations (4.32-fold differences between regions) and €60 825 pmp for LN hospitalizations (4.20-fold differences between regions). Large differences between regions were observed in the cost burden pmp relative to household income (4.70-fold for LN and 4.13-fold for SLE).

**Conclusion:**

In real-world clinical practice, the burden of in-hospital care of LN and SLE is heterogeneous across and within regional healthcare systems, offering the opportunity to benchmark best practice, optimize care and improve outcomes.

## Graphical Abstract

**Graphical Abstract:**
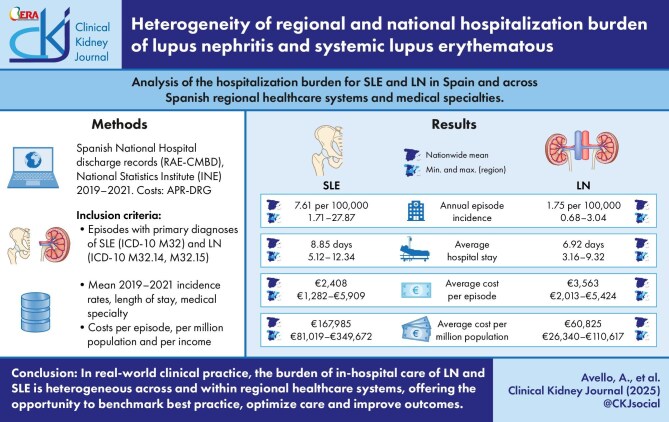

KEY LEARNING POINTS **What was known:**

- There is scarce contemporary information on nationwide burden and cost of hospitalization for lupus nephritis (LN) and systemic lupus erythematous (SLE) that addresses heterogeneity between regions and medical specialties.
- This information may be used to benchmark best practices

**This study adds:**

- The mean annual nationwide hospitalization case incidence in Spain was 7.61 and 1.75 per 100 000 population for SLE and LN, respectively, but regional differences were large, up to 48.0-fold for SLE and 6.9-fold for LN.
- There were also large regional differences in the distribution of cases between the three main specialties in charge of these hospitalizations—internal medicine, nephrology, and rheumatology—which are associated with differences in the length of hospital stay.
- The average cost per episode was €2408 for SLE and €3563 for LN, but there were wide differences in yearly costs per million population between regions (4.32-fold differences for SLE and 4.20-fold for LN).

**Potential impact:**

- An in-depth analysis of the drivers of heterogeneity regarding burden of hospitalization and cost of hospitalization for LN and SLE across regions and medical specialties may offer the opportunity to benchmark best practices, increase the sustainability of the healthcare system and improve outcomes.

## INTRODUCTION

Systemic lupus erythematosus (SLE) is a chronic relapsing autoimmune disorder that increases morbidity and mortality, mainly in young women [1]. Lupus nephritis (LN) is the most severe complication and may cause kidney failure requiring kidney replacement therapy [2, 3]. The incidence of LN is influenced by genetic background and socio-economic status. In the USA, for example, the incidence if LN is higher among African Americans than Caucasians [4]. In Spain, patients from Latin America have more severe SLE and a higher incidence of LN than those of European background [5]. Additionally, disease outcomes may be influenced by diagnosis timing, expertise and treatment protocols. Different specialists may be involved in the care of SLE and LN patients, adding further potential heterogeneity in clinical practice. Heterogeneity may be magnified when the healthcare system is fragmented. Spain has 17 different regional healthcare systems and wide regional differences in gross domestic product (GDP) per capita, healthcare resources, life expectancy and incidence of kidney failure [6, 7]. The population of regions (termed Autonomous Communities in Spain) ranges from 319 796 to 8 472 407, resulting in potentially vastly different levels of expertise in regional referral centres [8]. Similarly fragmented healthcare systems and regional differences exist in other countries [9].

National registries may be more informative regarding the overall burden and heterogeneity of care than experience from large referral centres [10–15], even if the latter involves multicentre studies. In Spain, the Rheumatology Society Lupus Registry (RELESSER) cohort only represented rheumatology departments [16, 17]. Furthermore, it did not address the heterogeneity of hospitalization burden related to SLE and LN across regions or specialties, which was also not presented in a wider analysis of 295 Spanish hospitals up to 2015 [18] or by similar exercises in England through 2015 [19], the USA in 2016 [20] and Taiwan through 2007 [21]. Thus there is scarce contemporary information on the nationwide burden of hospitalization for LN and SLE that is representative of most hospitals, not just large, specialized referral centres, and allows the exploration of heterogeneity in care without being marred by selection bias. Understanding heterogeneity in care may identify and benchmark best practices for timely diagnosis and adequate management. Benchmarking best practices may promote best clinical practices, reducing heterogeneity, optimizing care and improving outcomes [22]. Ideally, epidemiological analyses should include patients from all types of healthcare systems and socio-economic areas so that regions with small or impoverished populations are also represented.

Analysing Spanish data may be of interest for other high-income countries since, on top of fragmented public healthcare system, its demographic characteristics such as long life expectancy [longest in the European Union (EU) in 2022], low fertility rate (lowest in the EU after Malta) and large foreign-born population (third in the EU after Germany and France) are harbingers of changes to come in other countries [23–25].

To assess clinical practice and hospitalization burden heterogeneity for SLE and LN patients across regional healthcare systems, we analysed the 2019–2021 National Hospital Discharge Records database (RAE-CMBD) information from 644 public and private hospitals from all regions, as well as sociodemographic data from the Spanish National Institute of Statistics (INE). This novel, comprehensive and updated information disclosed high heterogeneity across and within regions regarding the incidence of hospitalization, duration, medical specialist and cost of hospitalization episodes, offering the opportunity to benchmark best practices and providing a model for similar exercises in other countries.

## MATERIALS AND METHODS

### Data sources

This is a retrospective study of the RAE-CMBD database. In 2016, the Spanish Ministry of Health established the compulsory *Registro de Actividad Sanitaria Especializada* (Specialized Care Registry – Minimum Basic Data Set) [26], expanding from a prior system that as of 2010 collected information from 295 hospitals [27]. The RAE-CMBD assembles administrative and clinical anonymized data for all hospital discharges from >90% of Spanish public and private hospitals (*n* = 644) from all 17 regions (termed Autonomous Communities) and the autonomous cities of Ceuta and Melilla. These data are open to researchers. Clinical data include diagnosis and procedures using the International Classification of Diseases, 10th revision (ICD-10) codes. Although RAE-CMBD contains data from 2016 onwards, uptake has been slow and hospital coverage only reached >90% in 2019–2021. Thus we retrieved data from the RAE-CMBD website [26] for patients discharged from hospitals between 2019 and 2021 with primary diagnosis codes M32 (SLE), M32.14 (glomerular disease in SLE) and M32.15 (tubulointerstitial nephropathy in SLE) to assess the burden of SLE and LN.

In the national databases there were no missing values, thus there was no need to address this methodologically by the authors. Any missing data prior to database release were handled directly by the INE using standardized imputation procedures [28, 29]. Specifically, prior to database release, the INE applied minor imputations for place of birth and nationality when information could not be retrieved from alternative administrative sources. For net household income, the INE used sequential multivariate linear regression models, implemented through the Imputation and Variance Estimation software, developed by Eurostat (Luxembourg). Although the specific imputation rate for the 2020 dataset is not publicly reported, in the most recent available edition of the Living Conditions Survey (2023), the imputation rate for this variable was 12.4%, and similar values were expected in previous waves.

Additional details are provided in the Supplementary Methods.

### Ethics

This research involved data obtained by accessing aggregated/unidentified information publicly and freely available in government databases (Ministry of Health: RAE-CMBD; Ministry of Economy: INE). Thus no ethical approval by the Instituto de Investigación Sanitaria de la Fundación Jiménez Díaz Ethics Committee was deemed necessary.

### Statistical analysis

The number of admissions for SLE and LN for each year (2019–2021) and by province and Autonomous Community was obtained from the RAE-CMBD and the mean number of annual hospitalizations over 3 years was used to calculate the annual incidence rate of LN and SLE hospitalization episodes per 100 000 population, using the mean population between 2019 and 2021 from the INE (Supplementary Table S1). Due to privacy concerns, the RAE-CMBD does not provide the precise number of hospitalization episodes when there are less than three per year (i.e. zero, one or two hospitalization episodes) per province. In these cases, we assigned one episode for that year and province.

Quantitative variables were summarized by mean and standard deviation (SD) or by median and interquartile range (IQR), according to the symmetry of the data distribution. The regional incidence of hospitalization was compared with the national incidence using Poisson regression models. The associations between incidence and regional characteristics (percentage of women, net household income, percentage of foreign-born population) were also assessed using univariable and multivariable Poisson regression models. The multivariable model was fitted with the three variables. The results of the models were summarized by relative risk, 95% confidence interval (CI) and *P*-value. Statistical analyses were performed using R version 4.3.1 (R Foundation for Statistical Computing, Vienna, Austria).

## RESULTS

### Heterogeneous incidence of hospitalization episodes for LN and SLE across regions

From 2019 to 2021, information was available for 66 262 724 hospitalization episodes nationwide covering 644 (>90%) Spanish public and private hospitals from all regions. Of these, 10 781 hospitalization episodes were for SLE, of which 2481 (23%) had a primary diagnosis of LN. Over 3 years, the mean annual number of hospitalization episodes was 3594 for SLE and 827 for LN (Table 1). Despite the COVID-19 pandemic, hospitalization episodes for 2019 and 2020 were in the same range, 3502 and 3403 for SLE and 790 and 727 for LN. Predominantly women were affected by both SLE (82%) and LN (83%). Hospitalization peaked at 12–44 years of age and then declined, with a steeper decrease in the 45- to 64-year range for LN than for SLE (Supplementary Fig. S1).

**Table 1:** LN and SLE all-hospitalization episodes in 2019–2021. All hospitalization episodes include conventional hospitalization, other hospital ambulatory modalities (including outpatient care), emergency room visits and at-home hospitalization. Data for individual regions (Autonomous Communities) ranked by population.

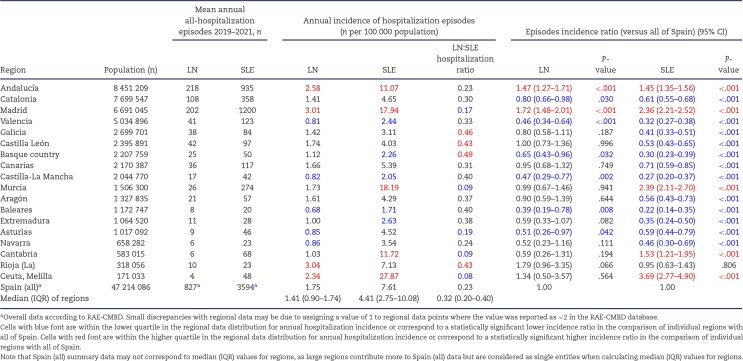

**Figure 1:**
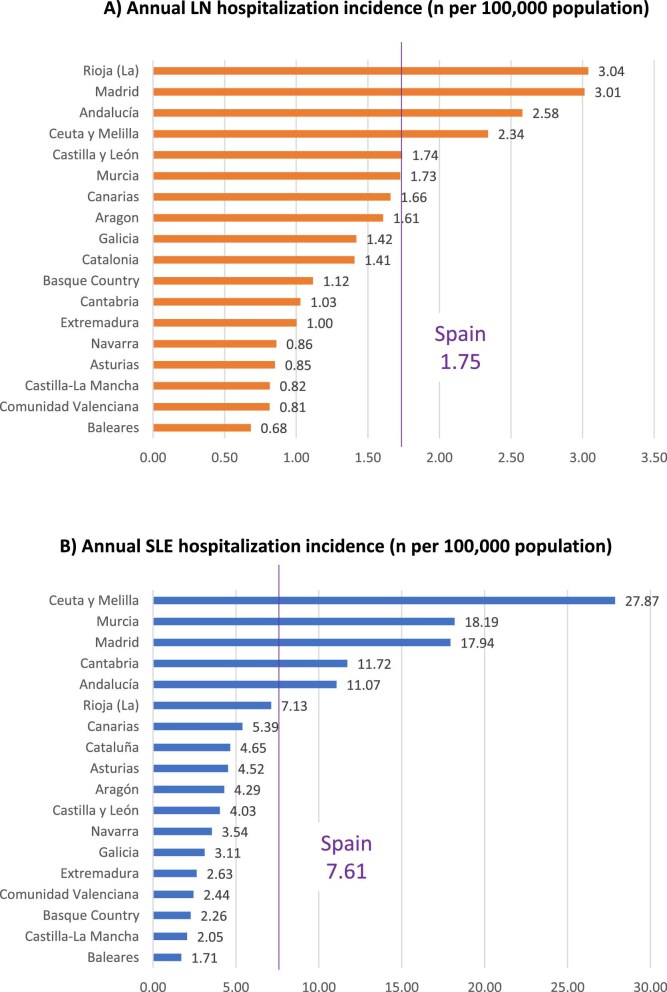
Incidence of **(A)** LN and **(B)** SLE hospitalization episodes in Spain in 2019–2021. Regions (Autonomous Communities) are ranked by incidence of (A) LN and (B) SLE hospitalization episodes. Data expressed as number per 100 000 population. The top four most-populated regions are Andalucia, Catalonia, Madrid and Valencia, which accounted for more than two-thirds of total hospitalization episodes. Note the consistency of ranking between LN and SLE hospitalization episodes in these heavily populated regions. Note the differences in scale between both graphs.

The annual nationwide hospitalization incidence was 1.75 per 100 000 for LN and 7.61 per 100 000 population for SLE. However, the range was wide: 0.68 (Baleares) to 3.04 (Rioja) per 100 000 and 1.71 (Baleares) to 27.87 (Ceuta, Melilla) per 100 000 for LN and SLE, respectively (Fig. 1), i.e. 4.5-fold and 16-fold differences, respectively. Two-thirds or more of hospitalization episodes occurred in the top four most populated regions—Andalucia, Catalonia, Madrid and Valencia—ranging from 0.81 (LN) and 2.44 (SLE) in Valencia to 3.01 (LN) and 17.94 (SLE) per 100 000 in Madrid (Fig. 1), i.e. 3.7-fold and 7.3-fold differences for LN and SLE, respectively. There were also wide differences between regions in the LN:SLE hospitalization ratio. Among the four most populated regions, it ranged from 0.33 in Valencia to 0.17 in Madrid, i.e. in relation to SLE hospitalizations, LN was a more common cause of hospitalization in Valencia or Catalonia than in Madrid.

**Figure 2:**
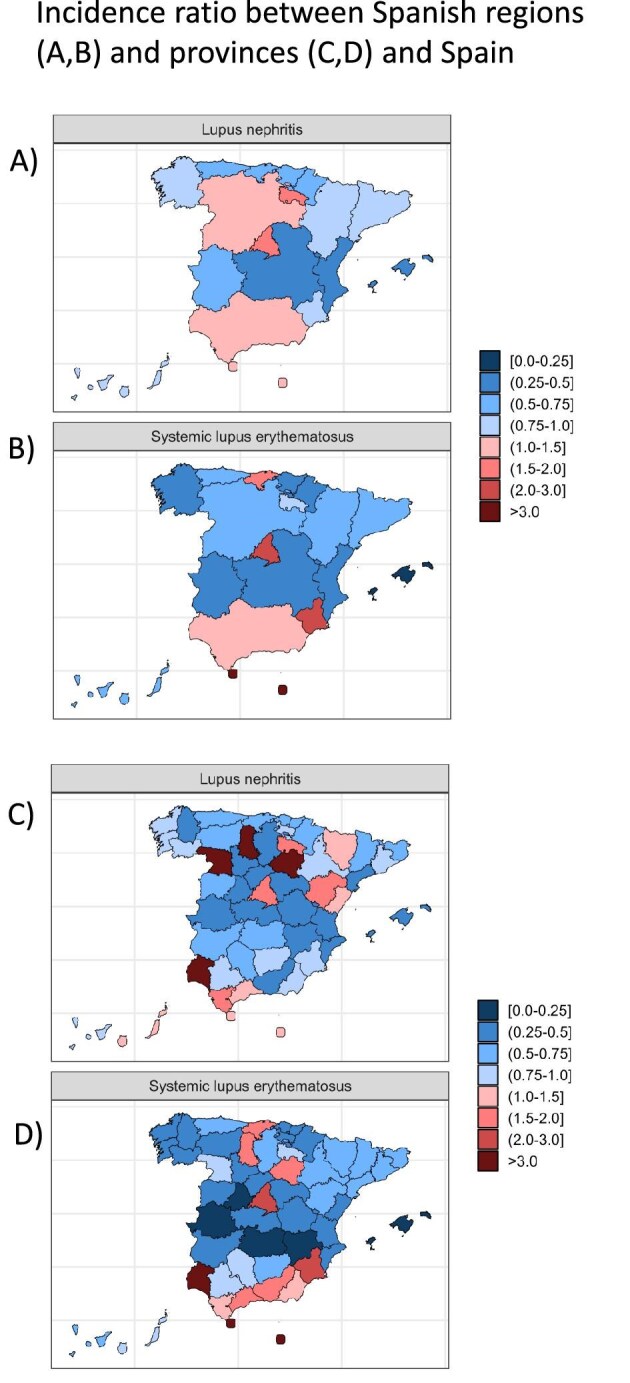
Choroplethic maps of incidence ratio for hospitalization in regions or provinces as compared with all of Spain in 2019–2021: **(A, C)** LN and **(B, D)** SLE. (A, B) Regions and (C, D) provinces.

The incidence ratio for hospitalizations between different regions and Spain ranged from 0.39 (95% CI 0.19–0.78) for Baleares to 1.79 (95% CI 0.96–3.35) in Rioja for LN (Table 1, Fig. 2A). The incidence ratio in eight regions differed significantly from all of Spain: in two highly populated regions (Andalucia and Madrid) it was significantly higher and in six it was lower, including Valencia. The heterogeneity was even larger for SLE. The incidence ratio for hospitalizations between different regions and all of Spain ranged from 0.22 (95% CI 0.14–0.35) in Baleares to 3.69 (95% CI 2.77–4.90) in Ceuta and Melilla (Table 1, Fig. 2B) and 17 of 18 regions differed significantly from all of Spain. Among the most populated regions, Andalucia and Madrid were again above the national mean for SLE hospitalization incidence, while Valencia and Catalonia were below.

Since clinical practice may change at the single hospital level, and some smaller provinces have just one main hospital and may present special socio-economic features, individual data from the 52 provinces may also provide insights into the heterogeneity of the burden of LN and SLE, although these data are marred by a lower number of events than regional data (Supplementary Table S2). Huelva [incidence ratio for LN hospitalization versus national mean 7.29 (95% CI 5.68–9.35) and for SLE hospitalization 4.16 (95% CI 3.56–4.85)] and Soria [incidence ratio for LN hospitalization 3.21 (95% CI 1.33–7.73) and for SLE hospitalization 1.62 (95% CI 0.90–2.94)] had the highest concordant incidence ratio of hospitalization for both conditions, while Baleares and Caceres (incidence ratio <0.45 and <0.25 for LN and SLE hospitalization, respectively) had the lowest. Ten provinces were concordant for incidence ratios that were significantly higher (Huelva, Cadiz, Málaga in Andalucia, Madrid and Soria in Castilla-Leon) or lower (Alicante and Valencia in Valencia, Toledo in Castilla-La Mancha, Vizcaya in Basque country, Asturias) than the national mean (Fig. 2C–D, Supplementary Table S2).

### Heterogeneity in duration and specialty of standard hospitalization episodes for LN and SLE across regions

In Spain, the mean duration of standard hospitalization episodes was 6.92 days for LN (Table 2). However, the range was wide, from 3.16 days in Aragón to 9.32 days in Navarra. Among the four most populated regions (Andalucía, Catalonia, Madrid and Valencia), hospitalization ranged from 6.89 days (Madrid) to 8.19 days (Andalucía).

**Table 2:** Distribution of specialties and stay length for LN and SLE hospitalization. Data expressed as % of all hospitalizations. Only internal medicine, nephrology and rheumatology were considered.

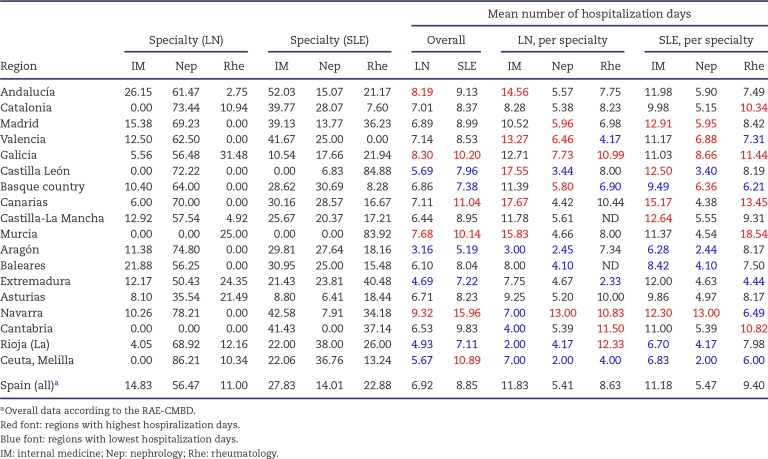

Most hospitalizations for LN and SLE occurred in nephrology, internal medicine and rheumatology.

LN was more commonly hospitalized in nephrology (56% of all LN hospitalizations), followed by internal medicine (15%) and rheumatology (11%) (Table 2). The range was from <1% in Cantabria and Murcia to 86% (Ceuta-Melilla) of LN hospitalizations in nephrology. For internal medicine the range was <1% (Catalonia, Castilla-León, Murcia, Cantabria and Ceuta-Melilla) to 26% of all LN hospitalizations (Andalucía). For rheumatology, the range was from <1% in nine regions to 31% of all LN hospitalizations in Galicia.

In Spain, the mean standard hospitalization for SLE was 8.85 days (Table 2) (range 5.19 days in Aragón to 15.96 days in Navarra). Among the four most populated regions, the range was from 8.37 days (Catalonia) to 9.13 days (Andalucía).

SLE was more commonly hospitalized in internal medicine (27.83% of all SLE hospitalizations), followed by rheumatology (22.88%) and nephrology (14.01%). Regions ranged from <1% of all SLE hospitalizations in nephrology in Murcia and Cantabria to 38% in Rioja. For internal medicine the range was <1% in Murcia and Castilla-León to 52.03% in Andalucía. For rheumatology, the range was <1% in Valencia to 84% in Castilla-León.

Some regions were consistently represented within the lowest quartile of hospitalization days for any specialty and cause of hospitalization (e.g. Aragón, Baleares and Ceuta-Melilla) while others were consistently represented among the highest quartile (Madrid, Galica and Canarias) (Table 2).

Heterogeneity for hospitalization characteristics was larger at the province level, even within the same region (Supplementary Table S2). LN hospitalizations in nephrology ranged from 39% (Granada) to 85–86% (Cadiz, Sevilla) in Andalucía and from 68% (Girona) to 100% (Lleida, Tarragona) in Catalonia. For SLE in Andalucia, internal medicine hospitalizations ranged from 13% (Seville) to 77% (Malaga), rheumatology hospitalizations from 3% (Huelva) to 69% (Cordoba) and nephrology hospitalizations from 3% (Granada) to 34% (Huelva). In Catalonia, the range was 35% (Barcelona) to 62% (Girona) for internal medicine, 10% (Girona) to 33% (Tarragona) for rheumatology and 20% (Tarragona) to 33% (Barcelona) for nephrology.

**Figure 3:**
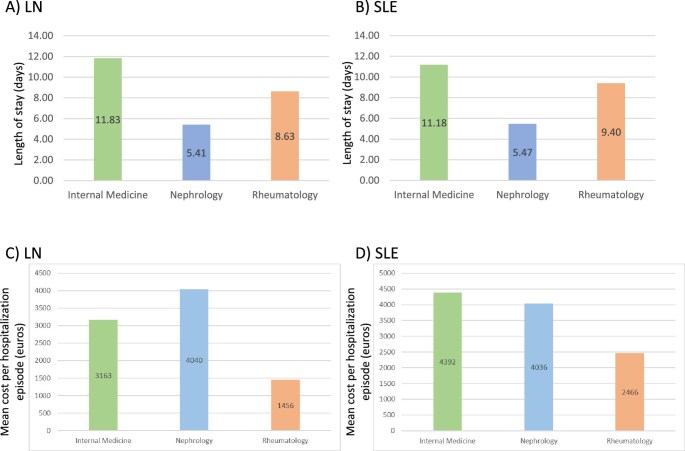
Length and cost of hospitalization for different specialties. **(A)** LN length of hospitalization in days and cost of hospitalization in euros per episode. **(B)** SLE length of hospitalization in days and cost of hospitalization in euros per episode. Data presented are for all of Spain for 2019–2021.

The mean hospitalization duration was also heterogenous across specialties (Table 2). Overall, hospitalization was shorter in nephrology and longer in internal medicine for LN or SLE (Fig. 3).

### Factors associated with hospitalization for LN and SLE

Table 3 shows demographic and economic factors associated with the incidence of hospitalization for LN or SLE. In multivariate analysis, higher net household income and a larger foreign-born population of European or North American origin remained associated with a higher incidence of hospitalization for LN or SLE, while a foreign-born population of African or Central American descent was associated with a higher incidence of hospitalization for SLE (Table 3B).

**Table 3:** Factors associated with the incidence of LN and SLE hospitalization episodes in Autonomous Communities in 2019–2021.

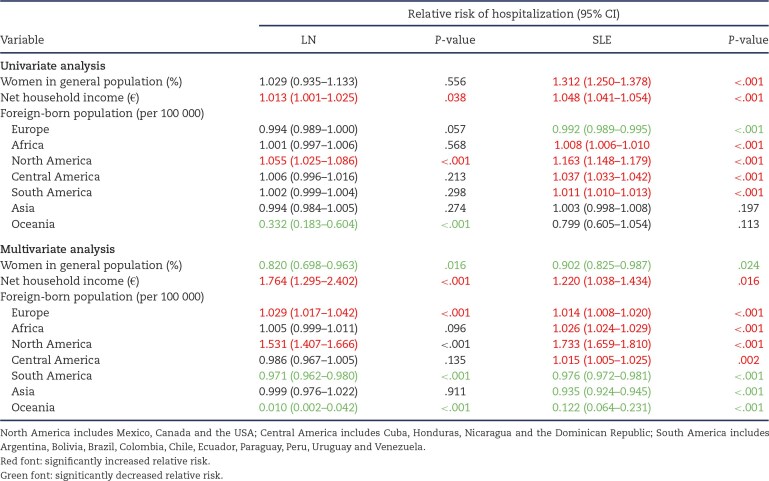

### Cost and budgetary impact of hospitalization for LN and SLE

Costs were calculated for the combination of standard hospitalization and other ambulatory modalities, as they are the only modalities covered by APR-DRG costs.

**Figure 4:**
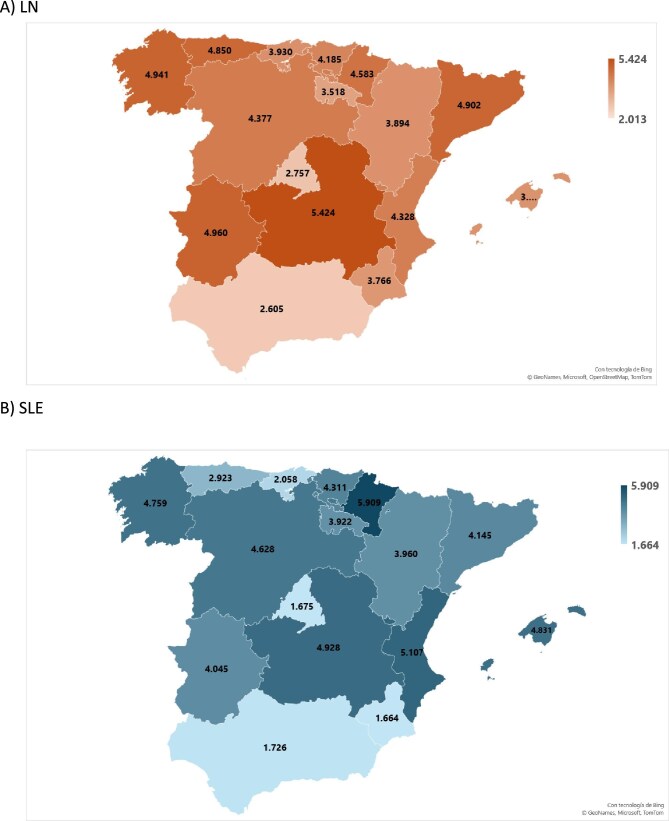

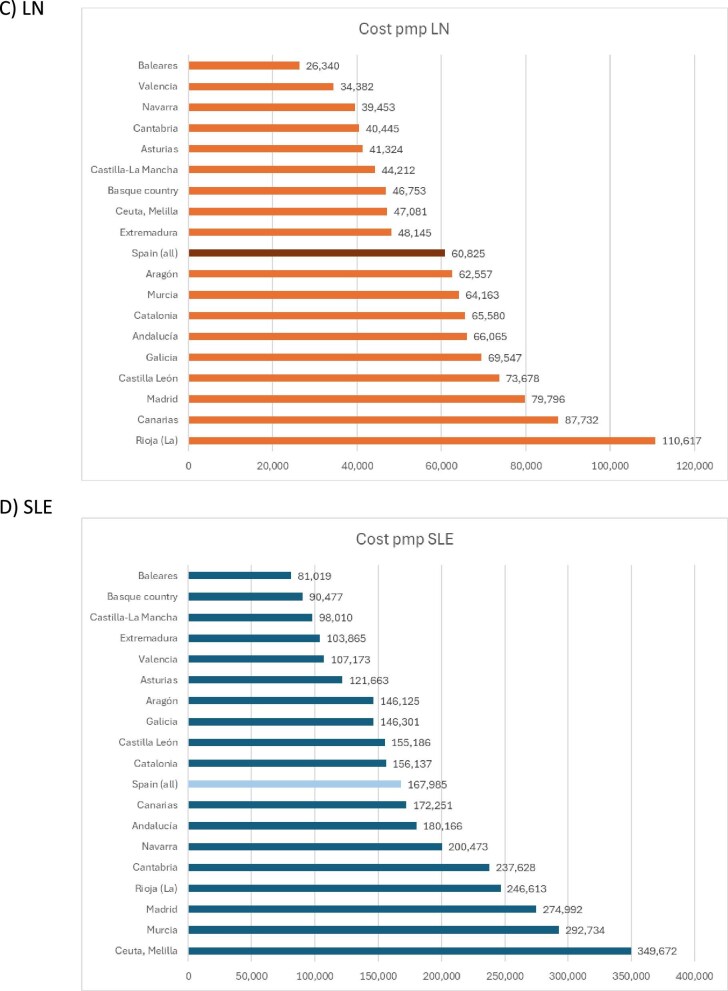

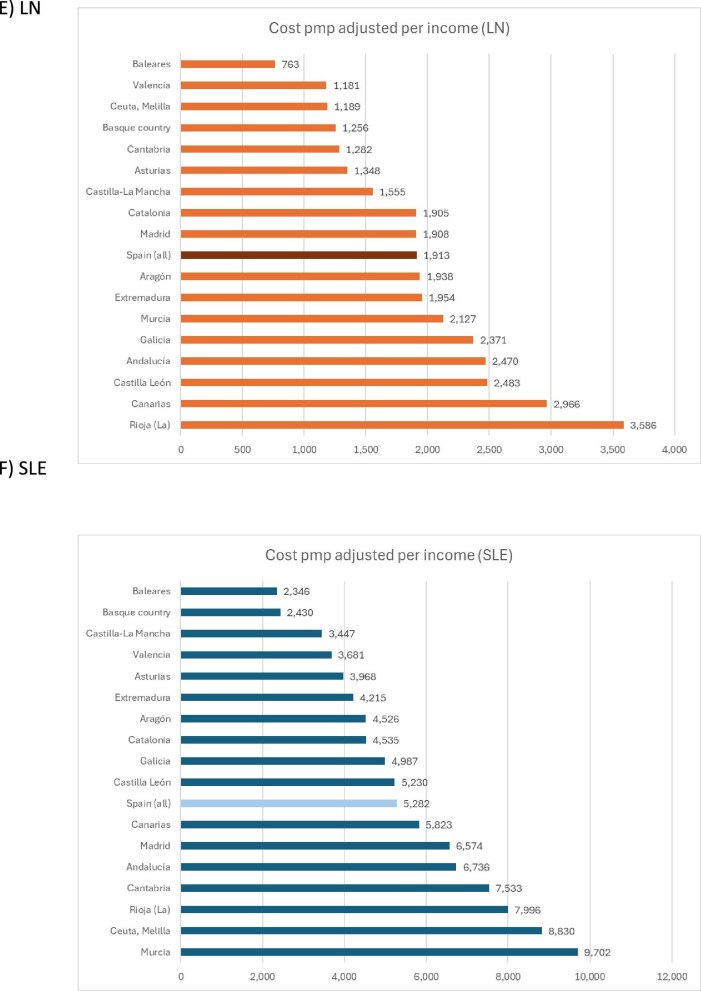
Hospitalization costs. Choroplethic maps for mean APR-DRG cost in euros per hospitalization episode for **(A)** LN and **(B)** SLE hospitalization in each region. Costs per million population where APR costs of all annual episodes in a region were normalized for the population of that region for **(C)** LN and **(D)** SLE hospitalization. Costs per million population per income where APR costs of all annual episodes in a region were normalized for the population of that region and then for the regional household income for **(E)** LN and **(F)** SLE hospitalization. Costs were calculated for the combination of standard hospitalization and other ambulatory modalities, as they are the only modalities covered by APR-DRG costs.

The average APR-DRG cost per hospitalization episode was €3563 for LN and €2408 for SLE, although there was regional heterogeneity (Table 4; Fig. 4A–B). The mean cost was also heterogeneous across specialties, being higher for nephrology and lower for rheumatology for either LN or SLE (Fig. 3C–D).

**Table 4:** Cost burden of LN and SLE hospitalization episodes in 2019–2021. APR-DRG costs were available for the combination of conventional hospitalization and other hospital ambulatory modalities (including outpatient treatment). Data per region (Autonomous Community), ranked by population.

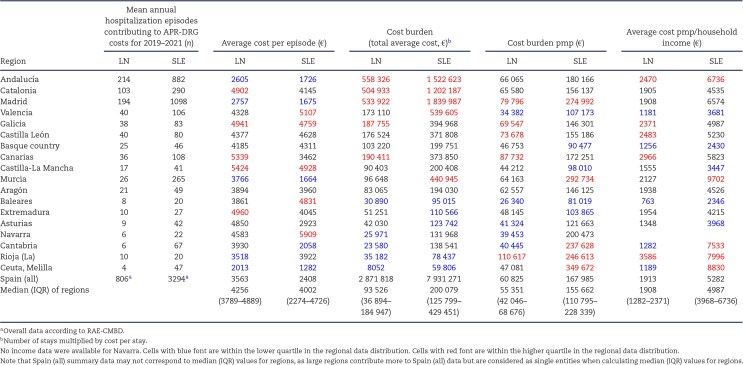

The average yearly costs were €2 871 818 and €7 931 271 in all of Spain for all LN and SLE hospitalizations, respectively (Table 4). It ranged from €8052 in Ceuta-Melilla to €558 326 in Andalucía and from €59 806 in Ceuta-Melilla to €1 839 987 per year in Madrid for LN and SLE hospitalizations, respectively (Table 4, Supplementary Fig. S2). Supplementary Table S3 shows province data.

Adjusted for pmp, the average yearly cost for LN and SLE hospitalizations was 3.02-fold and 3.39-fold higher for Madrid than for Baleares (Table 4) (Fig. 4C–D).

Relative to average household income (INE data available in Supplementary Table S1), annual LN hospitalization costs were highest in Rioja (€3586 pmp/€1000 of household income) and lowest in Baleares (€763 pmp/€1000 of income). For SLE, it was highest in Murcia (€9702 pmp/€1000 of income) and lowest in Baleares (€2346 pmp/€1000 of income). The range between regions was 4.70-fold for LN and 4.13-fold for SLE (Fig. 4E–F).

## DISCUSSION

The main findings are that there is marked heterogeneity in the burden of hospitalization for LN and SLE, specialists in charge of hospitalization, length of hospitalization stay and cost burden across and within Spanish regions. The originality and timeliness of the study is supported by its nationwide scope and analysis of recent years.

Prior studies have addressed hospitalization at the national level for SLE in different countries, including England through 2015 [19], Spain through 2015 [18], the USA in 2016 [20] and Taiwan through 2007 [21]. However, we did not find studies that addressed hospitalizations for LN separately or heterogeneity within countries or between specialties. Thus, reported nationwide data did not reflect potential heterogeneity or represent the current reality of LN and SLE hospitalization, given advances in therapy for SLE reflected by recent and rapidly updated guidelines [30–32]. Other studies reflected a single county or region data [15, 33] or single referral centres or regions and may not be representative of the wider SLE population or of real-world experience and practices [10–15].

Heterogeneity in methods between studies conducted in different countries may be explained in part by differences in health systems and SLE registries. Most studies report a similar proportion of women in hospital admissions (80–90%) and a similar median age (≈40–50 years) [15, 20, 34]. Several European studies and one Taiwanese study report similar lengths of hospital stay (≈9 days), however, in the USA it was significantly shorter (5–6 days) [20, 33]. A Danish study reported a mean duration of hospital stay of 6.4 days, however, it included outpatient hospital episodes [35], unlike our study, where the calculation of hospitalization days referred only to hospitalization, not to outpatient hospital episodes. It is possible that the use of outpatient hospital episodes differs for specialty. In this regard, various intravenous immunosuppressive treatments such as cyclophosphamide and rituximab have long been used to treat LN and may be administered on an outpatient basis [34, 36].

Few studies reported hospitalization incidence on the national level. A recent study in England reported a rate of 9.04 hospitalizations for SLE per 100 000 in 2014–2015 [19], which is higher than for our data but was lower than for some large regions such as Madrid and Andalucia. In a US study, LN was the main cause (32.45%) of hospitalizations for SLE as the primary diagnosis [20], slightly higher than for the current report and potentially reflecting differences in healthcare systems and genetic background, among other possibilities.

A prior study in Spain addressed hospitalizations for different causes in patients with SLE from 1997 to 2015, using an older database that had less hospital coverage and did not report on regional or specialty data [18]. The mean age (46.5 years) and female population (83.3%) was aligned with our more contemporary data. However, the average stay was longer (9.1 days), although it decreased over time, which would be consistent with shorter stays in our contemporary study: the most recent hospital stay (2011–2015) of 8.5 days was close to our observed stay of 6.92 days. However, the number of hospitalization episodes per year was higher in our analysis, likely related to the wider hospital coverage of our data.

The reasons behind the heterogeneity in hospitalization incidence, duration and costs cannot be assessed from the available data. It may represent real differences in incidence (different individual patients requiring hospitalization) and/or severity (some individual patients requiring multiple hospitalizations because of severe disease) of LN and/or SLE. This, in turn, may be related to different characteristics of the population, as suggested by the association with the regional prevalence of people born in certain world regions known to have a higher incidence of SLE. In this regard, Spain underwent rapid demographic changes in the 21st century. In 2024, Spain had a foreign-born population of 18%, up from 3% in 1999, mostly younger people and concentrated in regions with higher GDP per capita [37–39]. Most foreign-born people come from Spanish-speaking America and from North Africa. Additionally, the association of richer regions (net household income) with a higher incidence of hospitalization may be in line with the higher frequency of SLE in urban areas [40], although alternative explanations are possible. Thus richer regions also have a higher percentage of foreign-born young women.

Heterogeneity may also represent different local clinical practice regarding reasons for hospitalization and/or effectiveness or safety of local treatment protocols. As an example, a US study found significant state-level variation in readmission rates for SLE even when adjusted for differential case mix, suggesting that areas with a higher concentration of SLE-specialized centres and a higher level of clinical expertise might have better outcomes [32]. Additionally, clinical practice may differ between specialties, which may be influenced by local key opinion leaders and specialty-specific guidelines, given the existence of regional, national and international scientific societies [41–45]. An analysis of the drivers of these differences may also disclose dysfunction of the healthcare system as a whole: suboptimal access to primary or specialized care may delay diagnosis or treatment, increasing the likelihood of severe disease requiring hospitalization.

Additional factors may have influenced the great heterogeneity found in hospital stay length and associated costs. For example, the shorter hospital stay in the nephrology specialty could be related to its focus on procedures (e.g. kidney biopsies) or treatment protocols (periodic infusion of immunosuppressive drugs), while the longer stay in internal medicine could be explained by care for more complex presentations or complications. Local protocols for the administration of immunosuppressants, such as full hospitalization versus administration on an outpatient basis, may have also contributed. Differences in age, ethnic group, education and socio-economic level, CKD stage (in cases of LN) or comorbidities (diabetes mellitus, arterial hypertension) and the degree of therapeutic adherence could have influenced costs, length of stay and rehospitalization rates. Unfortunately, only aggregated data are available in the database for age and the other data are not available.

Inequity was found in the cost burden of hospital care for SLE when adjusted for pmp, which was even more evident when adjusted for household income in the different regions. This information is useful for healthcare planners, as it may help address the causes, benchmarking and assigning of resources. Moreover, it represents a paradigm that may be applied to other rare diseases.

Several limitations should be acknowledged. Some are inherent to nationwide registries and may include heterogeneous reporting. Data from smaller regions may be affected by the low incidence of a rare disease. However, evidence for heterogeneity in care was also found when comparing the most populated regions having a higher case load. Although they provide the most complete vision of in-hospital LN and SLE care so far in Spain, the data may not be generalizable to other countries or continents. However, the general concept that heterogeneity may exist, and that it is worth exploring, is likely generalizable to other contexts. The analysis did not provide insights into the underlying reason for the different lengths of hospitalization in different specialties. Despite the COVID-19 pandemic starting in 2020 and extending into 2021, there was no apparent heterogeneity in the number of hospitalization episodes during the pandemic.

The study has strengths. It analysed a nationwide registry in which all provinces and regions, large and small, with higher and lower income as well as private and public hospitals are represented, thus providing a complete picture of the burden of LN and SLE hospitalization. Additionally, it reflects data from the key specialties treating these patients—internal medicine, nephrology and rheumatology—and thus improves over potentially biased data generated by one or two specialties or a single or few referral centres. Finally, by averaging data from 2019 to 2021, it provides an updated vision of the healthcare burden, heterogeneity and costs of SLE- and LN-related hospitalizations.

In conclusion, the burden of LN and SLE hospitalization care is heterogeneous across and within regions regarding the incidence, duration, specialists and cost burden of admissions. This proof-of-concept study opens the door to further studies in other countries. Acknowledging heterogeneity in care is the first step to establish benchmark best practices to decrease clinical practice heterogeneity, optimize care and resources and improve outcomes. Assessment of heterogeneity in the burden of LN and SLE should be incorporated into future studies of the epidemiology and impact of these conditions.

## Supplementary Material

sfaf162_Supplemental_Files

## Data Availability

The data underlying this article will be shared upon reasonable request to the corresponding author.
