# Supplementary material for: Heterogeneity of regional and national hospitalization burden of lupus nephritis and systemic lupus erythematous
Source: Clin Kidney J. 2025 Jul 1;18(7):sfaf162. doi: 10.1093/ckj/sfaf162 (PMC12230550; doi:10.1093/ckj/sfaf162)
Supplement: sfaf162_Supplemental_Files [file sfaf162_supplemental_files.zip › Supplementary methods con S30.docx]

**Supplementary methods**

**Heterogeneity of regional and national hospitalization burden of lupus nephritis and systemic lupus erythematous**

Alejandro Avello^1,2^, Raúl Fernández-Prado^1,2^, Daria Abasheva^1,2^, Ignacio Mahillo^3^, Miguel Ángel González-Gay^4^, Catalina Martín-Cleary^1,2,5,6^, José Miguel Arce-Obieta^6^, María Vanessa Pérez-Gómez^1,2,5^, Beatriz Fernández-Fernández^1,2,5^, Alberto Ortiz^1,2,5^

^1^ Nephrology and Hypertension, IIS-Fundacion Jimenez Diaz UAM, Madrid, Spain

^2^ RICORS2040, Madrid, Spain

^3^ Statistics, IIS-Fundacion Jimenez Diaz UAM, Madrid, Spain

^4^ Rheumatology, IIS-Fundacion Jimenez Diaz UAM, Madrid, Spain

^5^ Department of Medicine, School of Medicine, Universidad Autónoma de Madrid, Spain

^6^ Health Information and Clinical Coding Department, IIS-Fundacion Jimenez Diaz UAM, Madrid, Spain

**Data sources**

Spanish Autonomous Communities may contain one or several provinces and have independent public healthcare systems free at the point of care. However, there are differences in public-private partnerships, timing to reimbursement for novel EMA-approved medications, or vaccination calendars, and others. Ceuta and Melilla share an independent healthcare system and for the purpose of this analysis will be considered a separate region. There is also heterogeneity in key population health indicators. As examples, life expectancy at birth in 2022 ranged from 79.71 in years in Ceuta to 84.76 years in Madrid(<https://www.ine.es/jaxiT3/Datos.htm?t=1448>) and the incidence of kidney failure requiring kidney replacement therapy, from 113 per million population (pmp) in Castilla-La Mancha to 192 pmp in Asturias(<https://www.senefro.org/contents/webstructure/SEN_2023_REER_V2_1_.pdf>).

RAE-CMBD provides aggregated data for hospitalization episodes, such as age, percentage of women, mean number of hospitalization days per hospitalization episode, the medical specialty at discharge, and mean number of hospitalization days per medical unit. RAE-CMBD includes ICD clinical coding for Primary and Secondary Diagnosis and Procedures, and the All Patient Refined Diagnostic Related Groups (APR-DRG) for each hospitalization episode. APR-GRD is a patient classification system that classifies hospital inpatients according to their reason for admission, severity of illness and risk of mortality, and is only valid for standard hospitalization and day hospital. Each APR-GRD group has an averaged and weighted cost assigned. The cost of a given hospitalization episode is estimated by multiplying the average APR-GRD cost for the group by the Severity of Illness indicator. RAE-CMBD provides the average cost per hospitalization episode (standard hospitalization and “Other ambulatory modalities”), estimated as the weighted average of the average DRG costs of all cases of a given process, calculated by multiplying the number of cases of each GRD and Severity Level by its average cost and dividing by the total number of cases of said process. The APR-DRG version for these years was v.36 and GRD 346 (connective tissue disorders).

RAE-CMBD included episodes of hospital utilization for standard hospitalization, other ambulatory modalities (this includes day hospital and other complex ambulatory procedures), hospital emergency room visits and hospitalization at home. Mean hospitalization days are only provided for standard hospitalization episodes. Costs were calculated for the combination of standard hospitalization and other ambulatory modalities as they are the only modalities covered by APR-GRD costs.

INE provides population-based information, which includes demographical information such as percentage of women, ancestry, as the rate per 100,000 population of foreign-born people and their geographical origin by continent (mean of 2019-2021 data), and socioeconomical data, such as net income per household in euros for 2020. For the reporting of ancestry, America is divided in 3 regions: North America (Mexico, Canada, United States of America), Central America (Cuba, Honduras, Nicaragua, Dominican Republic), South America (Argentina, Bolivia, Brazil, Colombia, Chile, Ecuador, Paraguay, Peru, Uruguay, Venezuela).It is important to note that in The United States, population from Central America, South America, and Spain are considered Hispanics because of a shared culture and language. This term, thus, becomes meaningless in Spain. However, these populations have a vastly different genetic background from country to country, and include Caucasian, African and Native American ancestry. However, race is not available in medical records in Spain.
